# Alloying and Refining Gold for Dental Purposes

**Published:** 1871-05

**Authors:** Mintern J. Brown


AMERICAN JOURNAL

OF

DENTAL SCIENCE.

Vol. V. THIRD SERIES--MAY, 1871. No. 1.

### ARTICLE I.

### *Alloying and Refining Gold for Dental Purposes.*

By Mintern J. Brown, D.D.S.

Although the science of alloying and refining gold, may not be considered as exactly coming within the province of the dentist, but rather as belonging to the jeweler or gold-smith, still a knowledge of these, purposes in the course of al most every dentist's practice, will be found to be of practical value, and to one whose field of labor may lie beyond the scope of "Dental Depot" conveniences, almost if not wholly indispensable; so also with the system of reducing gold into plate, and of the manufacture of solder, they may be of little use to those who practice in large cities, procuring their plate of any required size and thickness, and solder of any degree of fineness, from the dental depots, and in return selling the scraps, clippings, filings, &c., but to those situated at a distance from the cities and dependent upon coin, or pure gold, for the material they may use, a knowledge of the system and of the appliances for carrying it into effect, are of the greatest importance. Although many substitutes for gold have been introduced, this article still holds the field against, and is superior to all competitors, for it possesses many properties which peculiarly adapt it to the purposes for which it is used in dentistry. Gold in its pure state is so soft and yielding, as to be seldom used by dentists in the labratory, it has therefore been found necessary to combine with it some other metal, or metals, that shall, while imparting to it the desired degree of strength, elasticity and hardness, not impair its malleability, pliancy and purity. This combining with it of other metals is called alloying; alloys in respect to their use being to all intent new metals possessing properties, and differing in many respects from the constituents of which they are composed.

The metals with which gold is most generally combined are silver and copper, and although it might seem from theory, that the purer metal, would be less likely to increase the liability of gold to tarnish, while the baser would impart to it a disagreeable metallic taste. Still this is not warranted by experience, or facts, and gold when alloyed with copper unless reduced entirely to much, is as capable of resisting the action of acids, and is as free from any disagreeable metallic taste, as when alloyed with silver, beside it renders it more susceptible of a high and beautiful finish. The effect of these two metals upon gold are in strong contrast, copper giving hardness and elasticity, and deepening the color into a red, while silver preserves the softness and gives a greenish tinge to the original color of the gold. Platinum is sometimes used for alloying gold, but owing to the affinity of the alloys for oxygen, it is readily acted upon by nitric acid, and the septic acids of the mouth will in most cases, in a short time, be apt to corrode or make the metal very brittle. The terms by which the fineness of an alloy is expressed, are, taking pure gold as a starting point, either unity, 11.24 or 1000. In the first the fineness is given in fractions, in the second by parts called carats, and in the third by decimals. The fineness of our American coin for instance, would be expressed in the first system by 9-10, indicating that it contains 1-10 alloy, and 9-10 pure gold ; in gold the second by 21.6, alloy 2 4-10 carats, pure gold 21 6-10

carats, and in the third by 800, sne hundred parts alloy, nine hundred pure gold. Gold alloys to be permanently worn in the mouth, should be sufficiently pure to resist, under all circumstances, any chemical change that might affect the health or comfort of the wearer. If the health of the wearer were uniformly the same, and the secretions of the mouth always in their normal condition, gold of a very low carat might be used and undergo no material change, but beside the corrosive agents constantly being taken into the mouth, the fluids of the mouth, otherwise innoxious, are rendered more or less acidulated by a variety of diseases. Evils of no small degree may be inflicted by the use of debased gold, even if the use of gold of a low standard of fineness were accompanied by no evil results, and it should not be used, as it becomes discolored by the buccal secretions of the mouth, imparts a disagreeable taste, and when worn a few years becomes brittle. Gold therefore to be permanently worn in the mouth, should seldom or never be of a less degree of fineness than nineteen carats, and in some cases it should exceed this in purity. When gold of an unknown fineness is to be converted into plate, it should be refined first, and afterwards properly alloyed. This will not be necessary in using coin of a known standard. The refining of gold, or separating from it foreign metals with which it may be contaminated, is performed either by what is designated as the "dry method" or the "humid." Acknowledge of these methods is of the greatest importance to the dentist, as in the laboratory it is impossible to prevent gold from becoming mixed with other metals, as platinum, silver, solder, steel from files, and lead and zinc from dies and counter dies. The refining of gold by what is termed the "dry method," is effected by the action of either oxygen, chlorine or sulphur on the base metals which the gold may contain, converting them into oxides, chlorides or sulphurets. The refining agents generally exployed for this purpose, are nitrate of potassa, which yields oxygen; chloride of mercury, which yields chlorine, and sulphuret of antimony, which yields sulphur; other compounds contain these elements, but those already mentioned, are generally preferred, as they contain them in abundance, are readily decomposed by heat, and do not materially interfere with the process of separation by adding troublesome components to the alloy. The manner of refining by this method is first to remove all traces of iron and steel from the fragments and filings, to be refined, by passing a magnet through them several times, then to place them in a clean crucible, coated on the inside with borax, cover with a piece of crucible or fire-clay slab, then place in a furnace on a bed of charcoal; small portions of borax may first be added, and when the metals are reduced to a fluid state, the refining agents are to be introduced, from time to time, in small quantities It is a matter of no small discrimination to determine what re-agent should be employed. If, for example, zinc is present in a small quantity, nitrate of potassa should be used, which yielding oxygen that has a great affinity for this metal, readily effects its separation by converting it into an oxide but when tin is present, the act of decomposition is better effected with chloride of mercury, or some other compound that parts with chlorine. The washing should be continued from half an hour to an hour, according as the alloy is coarse or fine, the crucible may then be removed from the fire and allowed to cool gradually, after which it may be broken, and the button of gold removed and seperated from the stag that surrounds it. But if, as is generally the case, the alloy contains several metals having distinct affinities, or is of an uncertain composition, this process will have to be repeated several times, first with one refining agent, and then with another, and becomes to a considerable extent necessarily experimental. For reducing an alloy to pure gold by what is termed the "humid process," the solvents commonly used are nitric, sulphuric, and nitro-muriatic or hydro-chloric acids. In this process, the alloy is first dissolved in one of the above mentioned acids, and as the desired effect is more readily and directly obtained by the latter or hydro-chloric acid, this is generally made use of. This converts all the metals into chlorides, the chloride of silver being insoluble, and having a greater specific gravity than the liquid, will settle in the bottom of the vessel in the form of a grayish powder, the chlorides of the other metals being soluble will remain held in solution; by washing and pouring off, after the chloride of silver has had time to settle to the bottom, the solution may be readily separated from it; the proto-sulphate of iron (common green copperas) is then used to precipitate the gold, while the other metals still remain in solution. There are various other agents beside proto-sulphate of iron, which may be used for this purpose, but this is generally employed, being in all respects equal to the others, while being cheaper and more readily obtained; the copperas is dissolved in pure rain water, and should be filtered and permitted to stand until perfectly clear, it is then to be added to the gold solution gradually. So long as a precipitate is formed, this precipitate of gold will be a brownish powder; this should be permitted to settle to the bottom of the vessel, where it may be washed after pouring off the solution. This precipitate may contain traces of iron which may be removed by digesting it in dilute sulphuric acid, and the result, if all has been properly performed, will be pure gold. To form gold into plates suitable for dental purposes, the first thing to be done is to reduce it into the form of an ingot, this is done by placing the gold properly alloyed in a clean crucible lined with borax, and perfectly fusing it in a furnace, it is then poured into an ingot mould of iron, or charcoal, if the mould be of iron it should be oiled or coated on the inside with lampblack by holding it over the flame of an oil lamp, or gas; yet, before the metal is poured, it often becomes necessary to break up and remelt an ingot several times, as it not unfrequently occurs that in pouring, the metals arrange themselves in the ingot according to their different specific gravities, those of greater density passing to the bottom, while the higher remain at the top. The next step is to reduce the ingot somewhat in thickness by placing it on some hard surface, as the face of an anvil, and subjecting it to repeated blows with a moderately sized hammer. During the process of forging, the piece should be frequently annealed by bringing it to a full red heat ; this should be continued until the piece is reduced to about half its original thickness. The process of rednction is now continued by passing the ingot between the highly polished rollers of a mill used for this purpose, the rollers being brought a little nearer together each time the metal is passed between them, and the pieces being frequently annealed ; by this means it will be spread out into a sheet of greater or less thickness. Should the piece either in hammering or rolling, crack, it must be remelted and a few pieces of borax with a little muriate of ammonia thrown in. As regards the thinness of these plates, no prescribed rule can be given that will answer in all cases, as certain conditions of the mouth require modifications in this respect ; usually, however, plates for entire upper sets should correspond in thickness to No. 26 guage, and for the lower to No. 24, and for partial sets to an intermediate number. Plates for clasps or stays should correspond with 20 or 22, unless the alloy has a small quantity of platinum, when a less amount of substance is required.

The process for making plate to be used for stays and clasps, is exactly similar to that already described : first, re ducing the alloy to an ingot, and then by hammering and rolling into plate. From frequent annealing the plate will become oxydized, this may be readily removed by placing it in dilute sulphuric acid.

Solder is an alloy by which several pieces of the same or different metals are united ; necessarily they must fuse at a point below the metals to be joined, and should be composed of such ingredients as possess a strong affinity for the substance to be united, and they should also be as fine as the materials to which they are applied will admit of.

Gold solder is made by the combination in specified proportions of gold, silver, copper, and sometimes zinc and brass. The metals employed in making gold solder, if they are not pure, should be separately refined ; unless this is done it will be difficult if not impossible to ascertain their relative purity, which of course must be known to secure the desired effect. The method of making solder is, first, to melt the gold in a clean crucible, with a little borax and copper. If zinc or brass are to enter into the composition of the solder, it should be added last, after the other ingredients have become thoroughly melted, the whole rapidly stirred for a moment and immediately poured into an ingot mould. A piece of charcoal will be found more convenient, and will answer better for making a small quantity of solder than a crucible. Solder containing a grain or two of zinc will flow at a low temperature, and have a fine gold color, but it is apt to impart to the mouth a brassy taste, and also to become brittle after long use, and is apt, as is also solder containing brass, to be so stiff and even brittle, as to be with difficulty hammered or rolled, and the difficulty is increased by its low fusing point, rendering it almost impossible to anneal without melting it. The process of reducing solder into plate, is performed in a manner precisely similar to that already described for reducing gold alloy into plate for base for artificial teeth, clasps, stays &c. The plate should be formed somewhat thinner than a plate for an entire upper dentures.

In the different works on "Dental Surgery" and "Mechanical Dentistry," are given many receipts for the manufacture of solders that are said to work well and flow smoothly, but almost every dentist who manufactures his own solder, has a formula of his own, which, in his opinion, at least, is superior to all others. The greatest difficulty in the use of solder, generally occurs from a wrong method of soldering, and not from any defect in the solder.

